# Determinants of Women’s Drug Use During Pregnancy: Perspectives from a Qualitative Study

**DOI:** 10.1007/s10995-020-02910-w

**Published:** 2020-08-04

**Authors:** Gitau Mburu, Sylvia Ayon, Samantha Mahinda, Khoshnood Kaveh

**Affiliations:** 1 Centre for Global Health Policy, University of Sussex, Falmer, UK; 2 Kenya AIDS NGOs Consortium, Nairobi, Kenya; 3 School of Public Health, Yale University, New Haven, CT USA

**Keywords:** Pregnancy, Drug use, Maternal, Harm reduction, Kenya, Africa

## Abstract

**Introduction:**

Drug use during pregnancy can have negative effects on maternal and child health. However, there is a dearth of data regarding drug use among pregnant women in Kenya, where illicit drug use is on the rise. In this paper, we report factors influencing women’s decisions to use drugs during pregnancy.

**Methods:**

In 2015, we conducted in-depth interviews and focus group discussions with 45 women who inject drugs and five key stakeholders involved in provision of services to people who use drugs in coastal Kenya. Inductive thematic analysis was conducted to draw out themes related to key determinants of drug use during pregnancy.

**Results:**

Four key themes emerged outlining determinants of drug use during pregnancy: (i) the use of drugs to cope with the stress of unexpected pregnancy, (ii) the continued drug use during pregnancy to manage withdrawal, (iii) the dual effect of pregnancy on drug use either as a facilitator or as a moderator of drug use, and (iv) the role of male intimate partner in influencing women’s drug use during pregnancy.

**Conclusion:**

Our paper reports women’s drug use during pregnancy and the factors influencing this phenomenon. To safeguard the health and well-being of pregnant women and their unborn children, there is a need for education and awareness raising, implementing couple-based harm reduction approaches to leverage on positive male influences, improving availability of drug treatment, and provision of family planning interventions for women who use drugs.

## Significance

### What is Already Known on this Subject

Although there is a significant amount of research about drug use during pregnancy, most of it has been generated in high income settings. There is a dearth of data regarding drug use among pregnant women in low and middle-income countries (LMIC), including in sub-Saharan Africa.

### What this Study Adds

As opposed to observations from developed counties, women in this study did not report stopping their use of drugs once they got pregnant. Our findings indicate a need to put in place interventions to mitigate drug use during pregnancy in coastal Kenya.

## Introduction

Women make up a third of the people who use drugs globally (Larney et al. 2015). A significant proportion of these women are of reproductive age, which means that drug use and pregnancy are likely to converge. Substances most commonly used during pregnancy include nicotine, alcohol, marijuana, and cocaine (Forray 2016). Opiates and methamphetamine have also been reported, but less commonly (Jones et al. 2013; Minozzi et al. 2013; Patrick et al. 2012). Drug use during pregnancy can have detrimental effects on pregnancy and child health outcomes (Howell et al. 1999; Wong et al. 2011). However, the precise nature and extent of poor neonatal outcomes depends on the type of drug involved (Wong et al. 2011).

Given the detrimental impact of drug use in pregnancy, scholars have explored reasons why women use illicit substances in pregnancy. Evidence from this research suggests that a combination of factors determine women’s drug use, ranging from personal reasons such as lack of self-efficacy and internal stressors (Latuskie et al. 2019; Yotebieng et al. 2016), social reasons such as identity (Meurk et al. 2014), and structural reasons such as lack of information, or inability to understand risk because of the way that it is communicated (Latuskie et al. 2019; Loxton et al. 2013). In a rare study that explored the use of alcohol during pregnancy in Kenya, Yotebieng et al. (2016) found that using it as coping mechanism, lack of screening from health care providers, and mixed advise from family and friends were all contributing factors.

Despite its wide breadth, a key gap in existing research related to substance use in pregnancy is that most has been generated in high income settings (Forray 2016). There is a dearth of data regarding drug use (besides tobacco and possibly alcohol) among pregnant women in low and middle-income countries (LMIC) (Forray 2016). The lack of research related to female drug use is problematic because it hinders the generation of appropriate policies and interventions (Ayon et al. 2017). In Kenya, research related to female drug use generally is only starting to accumulate, and as Yotebieng et al. (2016) assert, even less of it is concerned with the use of drugs during pregnancy.

Indeed, as noted by Ayon et al. (2017), limited attention has been paid to the broader sexual and reproductive health needs of women who use drugs in Kenya. This is not necessarily surprising given that harm reduction services in Kenya are nascent (Rhodes et al. 2016). It was not until 2013 that the Ministry of Health initiated harm reduction programs for people who inject drugs (NASCOP 2013). However, coverage of comprehensive harm reduction services, including methadone for drug users is still sub-optimal nationally (Rhodes et al. 2015). Up to 23% of injecting drug users report sharing needles or syringes, due to poor availability (Kurth et al. 2015).

In addition, recent research reported that female drug uses have unmet need for family planning (Mburu et al. 2018b), and have sub-optimal utilization of antenatal care while pregnant (Ndimbii et al. 2018). Therefore, recognizing that drug use can occur in the context of pregnancy—as has been reported in other countries (Black et al. 2012)—is an important step towards responding to maternal and reproductive health needs of female drug users in Kenya. This is particularly relevant given the documented stigma towards women who inject drugs in Kenya (Mburu et al. 2018a), including from health care providers (Ayon et al. 2017; Guise et al. 2016) which limits the access and utilization of health services by women who use drugs.

In addition, understanding reasons why women in Kenya may use drugs during pregnancy is crucial to designing programs that improve both the maternal, neonatal and child health. Because of the differences in repertoire of drugs used in different countries, which may determine the kinds of policies and services needed locally, conducting this research in specific contexts and locations is essential. For example, methamphetamine is commonly consumed in South Africa (Jones et al. 2011), while in east Africa, where cannabis has been widely available, consumption of opiates such as heroin is being increasingly documented (Kurth et al. 2015; Syvertsen et al. 2015). In this paper, we build on previous work by Yotebieng et al. (2016) who explored alcohol use in pregnancy, to report factors influencing women use of a wider variety of drugs during pregnancy.

## Methods

### Study Design

We conducted a qualitative study using a combination of in-depth interviews and focus group discussions (FGDs) with female drug users and key stakeholder in coastal Kenya. Qualitative research methods are well suited to identifying participants perceptions and reasons for their behaviors (Esterberg 2002). In this study, interviews were added onto the FGDs to provide complementary information: it was anticipated that participants may fail to disclose some information in group settings, or conversely, participants in live conversations with peers could divulge information that was concealed in interviews (Bryman 2012).

### Setting

This study was conducted in two Kenyan coastal towns of Mombasa and Kilifi. In these two towns, basic harm reduction services were being provided to injecting drug users by two community-based organisations (CBOs): REACH OUT in Mombasa and MEWA in Kilifi. These two sites were selected primarily to enable buy-in from these two organizations, rather than any anticipated differences in the phenomena being investigated.

As is the practice in other community-based harm reduction programs (Coyle et al. 1998), outreach workers from the two CBOs made regular contact with drug users in their own localities, providing them with information on HIV, HIV testing, basic sexual and reproductive health services, as well as clean needles and syringes. Outreach workers referred drug users to the CBOs in case they needed additional interventions such as temporary shelter, or first aid. Clients who needed advanced medical care were referred to nearby tertiary public facilities (Ayon et al. 2017). The following Table 1 summarises the services that were being provided by the two CBOs.

**Table 1:** Services provided at the study sites

Service domain	Outreach-based services	Services at drop-in centres	Referrals to other health facilities
HIV	Condoms, HIV testing and education	HIV testing and counselling services	HIV treatment, testing of hepatitis C and Tuberculosis
Harm reduction	Clean needle and syringes, alcohol swab, cotton wool	Addiction counselling, first aid for violence and overdose	Provision of opioid substitution therapy with methadone
Sexual and reproductive health	Information on family planning and provision of tampons and oral contraceptive pills	Pre-natal education, provision of short-term reversible contraceptives	Long-acting contraceptives, ante-natal care, and cervical cancer screening
Social and child-care services	Transport to drop-in centres and health facilities	Personal hygiene items, day shelter, diapers for babies	Referrals for post-rape and legal assistance

### Participants Recruitment

Participants were recruited through the two CBOs. Outreach workers from the two CBOs invited women who use drugs to participate in interviews or FGDs during their routine outreach. Women who accepted were screened for eligibility and scheduled for interview and FGDs. To be included, participants had to be over 18 years of age (to allow for independent consent); be within the reproductive age bracket of 18–49 years; and have injected drugs within the past 90 days. Apart from women, five key local stakeholders were chosen to provide additional information, in consultation with representatives from the two local CBOs. Key stakeholders were selected based on their role, expertise related to community-based services and policies related to drug use. Participant recruitment continued until data saturation was achieved. The final sample comprised 45 women who inject drugs and five key stakeholders involved in the delivery of services to drug users. The five stakeholders were a community health worker (n = 1), outreach workers (n = 2), a Ministry of Health official (n = 1) and an outreach manager (n = 1).

### Data Collection

Of the 45 women, 24 participated in interviews (12 at each site), and 21 participated in three FGDs (2 sessions in Mombasa and 1 session in Kilifi). Interviews and FGDs were facilitated by experienced researchers who asked open-ended questions related to socio-demographic information; past and current drug use history; experiences with contraception, pregnancy and pregnancy termination; reproductive and sexual health, HIV testing, current living arrangements, and incarceration histories. The interviews and FGDs covered similar topics. A brief, standardized set of questions was used to collect additional socio-demographic data at the end of interviews. The stakeholders participated in separately-scheduled in-depth interviews. Stakeholder interviews were semi-structured and focussed on policy and health system aspects related to injection drug use. All interviews and FGDs were conducted in Swahili or English, depending on participants’ preferences. All data collection took place in private rooms on the premises of the CBOs or in stakeholders’ offices. All interviews and FGDs were audio recorded and lasted between 45 and 60 min.

### Data Analysis and Reporting

Socio-demographic data were entered into Microsoft Excel to summarize socio-demographic and drug use characteristics. Interviews and FGDs were transcribed digitally and translated into English as needed. All transcripts were imported into Nvivo (QSR International Pty Ltd), which is a useful tool for qualitative analysis (Bazeley 2007). Interview and FGD data were analyzed together through an inductive thematic approach (Bryman 2012). Nodes were created in Nvivo and these nodes were then populated with preliminary codes constituting labelled fragments of text quotes from participants. Codes were refined iteratively and dynamically in keeping with factors affecting women’s drug use. Codes were then categorized to generate themes (Charmaz 2000). The reporting of this study follows COREQ criteria for reporting qualitative research (Tong et al. 2007).

### Ethical Considerations and Approval

The study protocol was approved by the National Commission for Science Technology and Innovation NACOSTI (ref: P/15/8861/4510). The study was performed in accordance with the ethical standards laid down in the 1964 Declaration of Helsinki and its later amendments. In addition, effort was made to ensure that at least two of the three interviewers were female, and participants were offered a choice to indicate preference for gender of their interviewer. Participation in this study was voluntary, and written informed consent was obtained from each participant prior to their participation. Participants were informed they could discontinue their participation at any time. Interviews were conducted in a private place, and confidentiality was safeguarded by assigning a unique code to each participant and securing all data digitally.

## Results

### Sample Characteristics

Participants were relatively young with the average age being 28.5 (range of 19–49). The average participant had low levels of education. Almost a fifth (18%) had no formal education, half (51%) had attended primary school, and 27% had attended secondary education. Only one participant had attended post-secondary education. Over half (53%) were single, while the rest either had a live-in partner (27%) or were married (18%). Commonest sources of income were sex work (29%), ‘hustling’ or begging (24%), and casual labour (16%). Over a quarter (27%) were homeless and over half (53%) had been imprisoned in their lifetime. Most had at least one child (82%), did not use contraception (69%) or attend antenatal care during pregnancy. Seven women had no living child at the time of interview although the reason for this was not explored.

On average, participants had used different substances and drugs for a cumulative eight and a half years but had only injected for the last two and a half years. The primary drug injected was heroin, which was used by 85% of participants. Heroin was used on its own by 27% of the participants, and in combination with other drugs by over half (58%) of the participants. Most of these combinations involved alcohol, cigarettes, cannabis (locally known as Bhang), Rohypnol (Flunitrazepam, a common date-rape drug with sedative and hypnotic effects), Khat (locally as Miraa, Khat is Catha edulis plant whose leaves contain a psychoactive alkaloid stimulant, Cathinone), solvents (glue), or cocaine. Cocaine was used as the primary drug by 13% of the participants, and 4% of them used it in combination with other drugs, especially heroin or Rohypnol. Overall, polydrug usage was common, with 60% of the participants using multiple substances.

### General Use of Drugs in Pregnancy

Data suggested that use of drugs—including injecting drug use—during pregnancy was common. Women described how they were smoking heroin in the form of a “cocktail,” which was rolled up in cannabis or cigarettes, and a significant number of participants conceived while they were smoking or injecting drugs: The following exchange with one of the participants illustrates drug use around conception:

Q: And at that time when you became pregnant, were you still using drugs?

R: Yes, I was still smoking. I hadn’t started to inject but I was smoking (Participant # 1, 26 years old, Mombasa).

In many instances, women who were already using drugs when they became pregnant continued to use them until term. In a typical response to whether she continued to use drugs when she realized that she was pregnant, a participant stated that she used drugs “until I delivered” (Participant # 10, 21 years old, Mombasa). There were few exceptions whereby women reduced their intake of drugs when they got pregnant, mentioning that they “tried to reduce the amount when I became pregnant”.

The commonest narrative however, was that pregnancy was a prompt, rather than a barrier to use of drugs. Asserting that she had used drugs throughout her pregnancy, a participant described how she started using drugs when she became pregnant:

I started when I first became pregnant, until the end (Participant # 6, 33 years old. Mombasa).

In a similarly typical example, when asked when she started using drugs, another participant explained that it was during her second pregnancy that she initiated drug use:

It was then that I started to try, that one [known as] …cocktail. (Participant # 4, 26 years old, Mombasa).

While several participants started using drugs when they became pregnant, our interest and intention was to understand the reasons why women decided to use drugs while expectant, which we focus on henceforth.

### Use of Drugs to Cope with the Stress of Unexpected Pregnancy

Data suggested that women used drugs as a coping mechanism for unplanned pregnancy. This was particularly noted among younger women. For these women, unexpected pregnancy was a source of stress, and personal hardship. Faced with unplanned pregnancies, women started to use drugs as a coping mechanism. Describing the importance of her pregnancy in contributing to her initiation of drug use, a 23-year old participant explained that even though she had been in a sexual relationship with a drug user, she hadn’t used drugs until she got pregnant. Asked if she was using drugs during the initial periods of her intimate relationship with a drug using partner she mentioned that “I had not started…then I got pregnant, and after that, I started to smoke heroin”. (Participant # 12, 23 years old, Kilifi). Among five women, the effect of unexpected pregnancy was especially pronounced since their partners did not offer any support. Some of the men abandoned the women once they learnt of their pregnancies, and it was common to hear that men “denied” having impregnated the women (Participant # 3, 26 years old, Kilifi). Faced with these stressful situations, some women made decisions to turn to drugs for relief.

### Pregnancy Intentions as a Moderator of Drug Use

In contrast to situations where pregnancies were unplanned, some women had planned to get pregnant, but moderated their drug use in the pre-conception period. Our findings showed that women’s decision to use or inject drugs was mitigated by their perception of its potentially deleterious impact on their ability to conceive. Women stated that they were aware that chronic drug use often caused amenorrhea and secondary infertility, which could prevent conception. With this information, some women deliberately reduced their drug use to regain fertility. Hence, a desire to conceive emerged as a significant moderator of drug use. In an illustrative example, a 27-year old woman described her lack of periods and difficulty in conceiving. Asked about what was causing this difficulty, she attributed her amenorrhea and inability to conceive to her ongoing drug use:

The way I see it is that it’s because of my current use of drugs. When I stop is when I might get to conceive again (Participant # 3, 27 years old, Kilifi).

This phenomenon of minimising drug use in-order to conceive was common among a clear majority of women who conceived deliberately. One woman said:

I can do family planning using drugs. That means there is a way I can reduce [my drug intake] and get a baby (Participant # 9, 34 years old, Mombasa).

Further exploration of this process suggested that this participant, who had four children, regularly adjusted her drug use as her main ‘contraceptive’ approach:

**Q:** So you used the same technique for all of them?

R: All, that’s the technique I use. They have a spacing of four years each and there is no child I have had without planning for.

Q: You planned for all of them?

R: Yes, I planned for them all (Participant # 9, 34 years old, Mombasa).

### Continued Drug Use During Pregnancy to Manage Withdrawals

Data suggested that some women, particularly older ones, were very experienced in managing their drug use. Once these women became pregnant in keeping with their plans, some tried to reduce their ongoing intake. While there were distinctive patterns whereby older women reduced their drug use in-order to get pregnant, data suggested that they didn’t completely stop drug use. Instead, they continued to use *in moderation* during pregnancy. This moderated use was designed to keep withdrawal symptoms away, rather than to get ‘high’: Asked if she continued to use drugs as usual after realizing that she was pregnant, the above participant responded:

Yes. Ah! I increasingly down sized [consumption]. Whenever I want to conceive there is a way I reduce it to a minimum, and if I get pregnant there is a maximum I use. I snort/sniff so that I get healed from withdrawal, but not to get high. (Participant # 9, 34 years old, Mombasa).

### Dual Roles of Intimate Partners in Facilitating or Limiting Drug Use

Among women who were in relationships when they got pregnant, the use of drugs during pregnancy was often mediated—for better or for worse—by their intimate partners. In several instances, intimate partners indirectly caused women to start using drugs. There were instances in which women commenced their engagement with drugs in the pursuit of intimacy, or to fulfil their perceived ideals of a good relationship with intimate partners, especially in the context of pregnancy.

Describing her relationship with her intimate partner who was using drugs, the 23-year old participant from Kilifi (mentioned previously), who associated her drug use with getting pregnant recounted how she started to use drugs in the hope that she would get along with her drug using intimate partner. When asked why she started using drugs during pregnancy, she said it was “because the person who impregnated me was selling [drugs]”. She went on to state that “I decided to use [drugs] with the hope that we could get along” (Participant # 12, 23 years old, Kilifi). Although hers was not applicable to all women, her situation illustrated the role that her intimate relationship played in her decision to start using heroin at a time when it was potentially harmful to the health of her unborn child. As the above case illustrates, participants’ use of drugs was prompted by the need to get along with the biological fathers of their children, who were using drugs. This was emphasized by a key stakeholder who noted that:

It is very easy for a woman who has a partner who is using drugs to be driven into drug use. It is very easy because the trust is very high upon the partner (Stakeholder # 1, outreach worker, Mombasa).

On a positive note however, data suggested that some intimate partners often supported their female partners to reduce their drug intake, particularly in the context of planned pregnancies. A participant who had 5 children described her regular decisions to reduce drug intake in-order to conceive, highlighting that she discussed and involved her intimate partner in those decisions:

He affirmed he will provide for the child, and then I told him I am ready even to stop. Then we left Kilifi and went to the interior village and after three months I got pregnant (Participant # 9, 36 years old, Kilifi).

Narrating the importance of this agreement to manage her dug use in order to conceive, the above participant remarked:

It is upon you both to stop [using drugs] so that you get a child; or one of you to stop so that you get a child (Participant # 9, 36 years old, Kilifi).

However, the complex relationship between women’s drug use, planned conception, and their intimate partners did not always result in reduced drug use during pregnancy particularly in the context of addiction. In some cases, intimate partners—particularly those who did not use drugs or who mainly smoked drugs—were against injecting of drugs during pregnancy, and this created rifts when women continued to inject. This was illustrated in the case of one participant who had planned her pregnancy, but could not abstain from injecting, contrary to her partner’s wishes. Asked if she continued to use drugs after getting pregnant and the how her partner reacted to ongoing drug user, this participant responded:

Yes, I just continued with the drugs…He had tried to help me by all means; he even brought me to this rehab and enrolled me for seven days. He had determination of not deserting me…He told me doesn’t want it but then I could not stay without using, then we had to call it quits. (Participant # 11, 33 years old, Kilifi).

This was particularly important given that drug treatment was rare in the study setting, yet the partner had made efforts to cater for it. Emphasizing the lack of harm reduction services in the study context, a stakeholder lamented how “drug users have been neglected for long, and addiction has now become a big issue” (Stakeholder # 1, outreach worker, Mombasa).

## Discussion

In this paper, we highlight drug use during pregnancy among women in Coastal Kenya. We report how drug use began or continued during pregnancy. Overall, a clear majority of women continued to use drugs while pregnant, and none reported stopping using them due to pregnancy. This finding is generally consistent with that reported by Yotebieng et al. (2016) showing that alcohol abuse was prevalent through-out pregnancy among Kenyan women. However, our study extends these findings by including women who use a wide variety of substances, including heroin and cocaine.

More broadly, the finding that women in our study initiated or continued to use illicit drugs during pregnancy is a departure from observations in developed counties. Research from high resource settings report that significant reductions in drug use occur between the second and third trimesters (Ashley et al.^2003^). Total abstinence from substance abuse is also commonly reported (Ashley et al. 2003). In contrast, we found that women in our study did not abstain from drug use when they got pregnant. While studies have shown that women’s concern regarding the impact of their drug use on the developing fetus motivates them to curb their substance abuse (Higgins et al. 1995), this did not emerge as a determinant of women’s drug use in our study.

This is not to mean that such concerns did not exist. Although these concerns may have intrinsically existed among some women, they may have been obliterated by other stronger drivers of continued drug use. Drug-using women often encounter significant environmental and socioeconomic stressors (Forray 2016). These stressors have been reported as contributing to substance use during pregnancy (Latuskie et al. 2019), including among Kenyan women (Yotebieng et al. 2016).

In our context, drug treatment is relatively rare (Mburu 2019), and many women resorted to using drugs to manage their withdrawal, particularly of heroin. Withdrawals may be responsible for the low levels of abstinence from drug use during pregnancy observed in our study, by overriding women’s natural cognitive motivation for a healthy baby and reducing their practical opportunities to limit their drug use during pregnancy. For this reason, we suggest that improving availability of drug treatment could mitigate ongoing drug use during pregnancy. Evidence shows that expanding drug treatment for pregnant women, for instance with methadone, is effective and feasible (Minozzi et al. 2013; Wong et al. 2011).

In our study context, social and community-based support services, such as educational and awareness raising activities, as well as parenting classes and workshops could be useful to inform women regarding the risks of drug use during pregnancy. There is a strong case to be made for the expanded use of community-based outreach to reach, screen, educate and refer pregnant women who use drugs to appropriate services. Screening of pregnant women for opioid use of by health care providers is generally recommended (Wong et al. 2011) but may not happen since most pregnant drug users in our context have limited or negative contacts with health facilities (Ndimbii et al. 2018), partly due to stigma (Ayon et al. 2017; Guise et al. 2016; Mburu et al. 2018a). Stigma from health care providers towards female drug users is particularly strong during pregnancy (Peters et al. 2003; Simpson and McNulty 2008), and therefore outreach services could be particularly useful in enabling pregnant women to mitigate it, by providing education and close support during pregnancy. The use of lay outreach workers to educate and screen has been successful in the context of alcohol abuse in other countries (O’Connor et al. 2014).

Given that many participants blamed unexpected pregnancy as an impetus for drug use, prevention and/or planning of pregnancy would be critical. Evidence from this study sample, published elsewhere, shows that the utilization of contraception among them is very low (Mburu et al. 2018b). This is consistent with research from developed countries showing that without interventions, women who use drugs such as opiates have high rates of unwanted pregnancies and low rates of contraception (Black et al. 2012; Weber et al. 2003). Thus, provision of family planning services to mitigate the effects of unwanted pregnancies and its associated shocks among drug-using women is essential in our study context.

Finally, our findings show that intimate partners had an influence on women’s drug use. This finding suggests that using a ‘couple approach’ would be useful to harness the positive influence of male partners that are keen to reduce their pregnant partner’s drug consumption. We suggest that motivational counselling to a woman and her male partner can unlock positive male influence to limit women’s drug use, especially during pregnancy. Other scholars have argued that incorporating couple-based approaches into substance abuse services can mitigate gender-specific drivers of female drug use (Ashley et al. 2003), including in pregnancy, by leveraging on positive role of male partners (Hammer 2019).

### Limitations

Our study was cross sectional in nature, conducted among a convenience sample of female drug users. These women were recruited through an outreach-based harm reduction program. Therefore, their health-related behaviors may differ from the behavior of those without such contact. Furthermore, it is possible that recall and social response bias may have affected our findings. In addition, we did not collect data on women who refused to participate, which may have introduced non-response bias. Nevertheless, our study provides useful data that could inform the development of interventions to mitigate drug use during pregnancy in the study context.

## Conclusion

Our paper has chronicled use of illicit drugs during pregnancy, a reality that makes a strong case for efforts to limit this phenomenon, in-order to safeguard the health and well-being of drug-using pregnant women and their children. To this end, we suggest that immediate critical actions should include: expanding educational activities, leveraging on couple approaches to harm reduction, improving availability of drug treatment, and expanding family planning interventions for these women.
